# TWO_2_ Therapy Demonstrates Clinically Meaningful Long-Term Outcomes Compared to Other Advanced Wound Care Modalities: Real-World Evidence Supported by Mechanistic and RCT Clinical Data

**DOI:** 10.3390/jcm15124780

**Published:** 2026-06-19

**Authors:** Anahita Dua, Naseer Ahmad, Cyaandi R. Dove, Matthew J. Regulski, Sara Rose-Sauld, Matthew G. Garoufalis

**Affiliations:** 1 Division of Vascular and Endovascular Surgery, Harvard Medical School, Massachusetts General Hospital, Boston, MA 02114, USA; 2 Department of Vascular Surgery, Manchester Vascular Centre, Manchester M13 9WL, UK; 3 Department of Endocrinology, Diabetes and Clinical Nutrition and the Harold Schnitzer Diabetes Health Center, Oregon Health & Science University, Portland, OR 97239, USA; 4 Ocean County Foot & Ankle Surgical Associates, P.C., Forked River, NJ 08753, USA; 5 Division of Podiatry, Department of Orthopedic Surgery, Massachusetts General Hospital, Boston, MA 02114, USA; 6 Department of Podiatric Medicine and Surgery, Western University of Health Sciences, Pomona, CA 91766, USA; 7 Professional Foot Care Specialists, Chicago, IL 60632, USA

**Keywords:** chronic wounds, topical oxygen therapy, intermittent compression, cyclical pressurized topical wound oxygen (TWO_2_), multi-modality intermittent topical oxygen therapy (ITOT), inflammation resolution, wound healing, advanced wound care

## Abstract

**Background/Objectives**: Chronic diabetic foot ulcers (DFUs) and venous leg ulcers (VLUs) remain a major source of morbidity, healthcare utilization, and limb loss, despite adherence to established standards of care protocols and the widespread availability of advanced wound technologies. Many advanced modalities only target isolated aspects of wound healing and fail to address the complex, interdependent pathophysiology of chronic wounds, particularly tissue hypoxia, edema, impaired microcirculation, and persistent inflammation. Cyclical Pressurized Topical Wound Oxygen (TWO_2_) therapy is a home-based, multimodal intervention that combines humidified topical oxygen delivery with cyclical non-contact compression to address these core drivers simultaneously. **Methods**: This review synthesizes mechanistic rationale and evidence from randomized controlled trials, long-term venous ulcer studies, and real-world comparative effectiveness analyses. Emphasis is placed on the large cohort study by Yellin et al., which directly compared TWO_2_ with other advanced modalities including negative pressure wound therapy (NPWT), skin substitutes, and growth factor therapies. **Results**: Across these studies, TWO_2_ therapy is consistently associated with improved healing durability, reduced recurrence, and substantial reductions in hospitalization and amputation rates compared with both standard care and advanced wound therapies. **Conclusions**: The convergence of randomized and real-world evidence supports TWO_2_ therapy as a clinically meaningful and mechanism-driven adjunctive treatment option for patients with chronic, high-risk lower-extremity wounds.

## 1. Introduction

Chronic lower-extremity wounds represent a growing global health challenge, driven by the increasing prevalence of diabetes, vascular disease, and advanced age [1]. Diabetic foot ulcers (DFUs) and venous leg ulcers (VLUs) are among the most challenging to manage, frequently resulting in serious complications such as infection, hospitalization, and amputation. These adverse outcomes contribute significantly to morbidity, mortality, and healthcare costs [2,3,4,5]. Despite the availability of numerous advanced wound care technologies, real-world outcomes remain suboptimal. Many wounds fail to progress beyond the inflammatory phase of healing, even when treated with advanced therapies, such as negative pressure wound therapy, bioengineered skin substitutes, or topical growth factors. A common limitation of these approaches is their inability to adequately correct the chronic wound microenvironment, which is characterized by hypoxia, edema, impaired microcirculation, and excessive protease activity [6,7,8]. Cyclical Pressurized Topical Wound Oxygen (TWO_2_) therapy has emerged as a promising adjunctive treatment modality capable of directly addressing oxygen deficiency while concurrently reducing edema, improving perfusion, and modulating inflammation.

This article synthesizes findings from randomized and real-world studies, contextualizes outcomes against other advanced treatments, and integrates mechanistic insights that provide the foundation for the clinically meaningful long-term healing outcomes observed with TWO_2_ therapy.

## 2. Materials and Methods

This is a narrative review of the mechanistic, randomized, and real-world evidence base for TWO_2_ therapy in chronic lower-extremity wounds. Literature searches were conducted in PubMed/MEDLINE (National Library of Medicine, Bethesda, MD, USA; https://pubmed.ncbi.nlm.nih.gov, accessed on 1 April 2026), the Cochrane Library (Cochrane, London, UK; https://www.cochranelibrary.com, accessed on 1 April 2026), and ClinicalTrials.gov (U.S. National Institutes of Health, Bethesda, MD, USA; https://clinicaltrials.gov, accessed on 1 April 2026) for studies published between January 2000 and March 2026, using the search terms: diabetic foot ulcer, venous leg ulcer, topical oxygen therapy, TWO_2_, ITOT, intermittent topical oxygen, wound healing, negative pressure wound therapy, hyperbaric oxygen therapy, skin substitutes, growth factors, and chronic wound. Inclusion criteria were peer-reviewed human studies in the English language with relevance to mechanistic rationale, comparative effectiveness, or long-term clinical outcomes. Studies were selected by the authors based on clinical relevance, methodological quality, and contribution to the evidence base. References were organized and managed using Mendeley Reference Manager (version 2.144.0; Elsevier, Amsterdam, The Netherlands; https://www.mendeley.com, accessed on 1 June 2026).

### 2.1. Pathophysiologic Rationale for Oxygen-Based Multimodal Therapy

Chronic wounds differ fundamentally from acute wounds in both biology and behavior. Rather than progressing through the orderly phases of healing, chronic wounds remain trapped in a state of persistent inflammation [9,10,11]. Tissue hypoxia is a defining feature of this state, resulting from macrovascular disease, microvascular dysfunction, edema-related capillary compression, impaired oxygen diffusion, and inflammatory metabolic demand [12].

Hypoxia disrupts multiple healing pathways. Fibroblast proliferation and collagen deposition are oxygen-dependent processes, as is angiogenesis. Immune function is similarly compromised, reducing bacterial killing, and increasing the risk of infection [12].

Edema increases interstitial pressure, compresses capillaries, and lengthens oxygen diffusion distance, further amplifying tissue hypoxia. Inflammatory mediators and proteases accumulate due to inadequate lymphatic clearance, degrading growth factors and extracellular matrix components [13,14]. Repetitive ischemia–reperfusion injury generates oxidative stress, further impairing tissue repair [6].

Effective chronic wound therapy must therefore address oxygen delivery, fluid dynamics, edema, and inflammation in an integrated manner. Therapies that target only wound size, exudate, or surface coverage may fail to produce durable healing if these underlying drivers are not corrected.

### 2.2. The Synergy of Topical Oxygen, Cyclical Compression and Humidification

Cyclical Pressurized Topical Wound Oxygen therapy was developed to address the multifactorial pathophysiology of chronic wounds through a single, integrated system. The therapy combines three synergistic components: topical oxygen delivery, cyclical non-contact compression, and humidification.

Topical oxygen delivery increases tissue oxygen tension at the wound surface, supporting oxidative bacterial killing, collagen synthesis, and angiogenesis [12,15,16,17,18,19,20]. Unlike systemic oxygen approaches, topical delivery targets the wound directly, minimizing systemic exposure while maximizing local effect [16].

**Figure 1:**
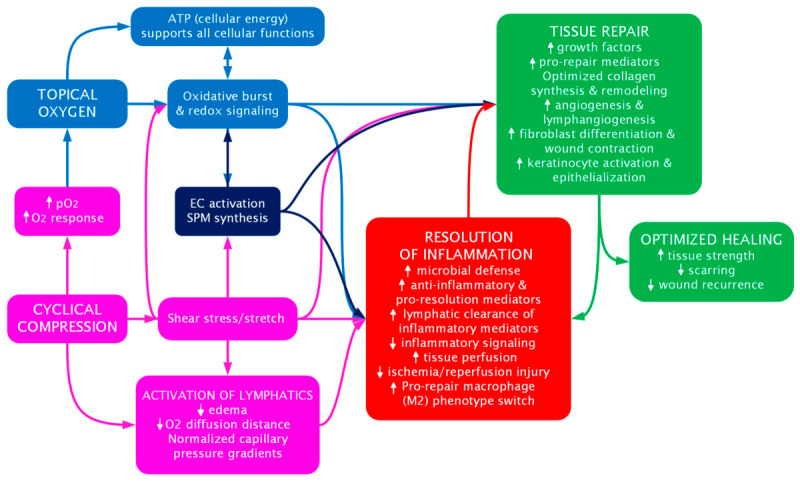
TWO_2_ Mechanism of Action: This figure presents a conceptual framework of the proposed integrated cellular and molecular mechanisms by which topical oxygen and cyclical compression synergistically promote wound healing. Pathways shown are derived from published mechanistic and clinical literature; the figure is not intended to depict quantitative relationships, and the modulating influence of variables such as wound age, severity, and comorbidity burden is recognized. Topical oxygen increases tissue oxygen tension to fuel ATP production, enhance microbial defense via oxidative burst, activate redox signaling, and optimize collagen synthesis and crosslinking. Cyclical compression increases the partial pressure (pO_2_) of topical oxygen and activates lymphatic function, improving clearance of inflammatory mediators, reducing edema, decreasing diffusion distance for oxygen, normalizing capillary pressure gradients, and restoring perfusion. Compression-induced shear stress and shear stretch activate endothelial cells (ECs) and stimulate the biosynthesis of specialized pro-resolving mediators (SPMs). This initiates a cascade of anti-inflammatory and pro-resolution signaling, including polarization of macrophages toward the reparative M2 phenotype and acceleration of inflammation resolution. In parallel, M2 macrophages, SPMs, and ECs upregulate growth factors and reparative cytokines that direct wound repair and remodeling. Activated ECs stimulate angiogenesis and lymphatic angiogenesis, while fibroblasts drive collagen synthesis, ECM production, and myofibroblast differentiation, enabling wound contraction. Keratinocyte activation promotes epithelialization, and during remodeling, fibroblast activity enhances collagen fiber organization. Collectively, these pathways promote efficient resolution of inflammation, improved perfusion, increased tissue strength, reduced scarring, and lower wound recurrence. pO_2_, partial pressure of oxygen; EC, endothelial cell; SPM, specialized pro-resolving lipid mediators; NADPH, nicotinamide adenine dinucleotide phosphate; ATP, adenosine triphosphate; M2, pro-repair macrophage phenotype (Illustration by Blakely MM, 2026, reprinted/adapted from ref. [6]).

Therapeutic level cyclical compression augments these effects by increasing the oxygen partial pressure delivered to the wound bed, facilitating lymphatic clearance of inflammatory mediators, reducing edema, restoring microvascular pressure gradients, and improving oxygen diffusion into hypoxic tissue [6]. At the cellular level, cyclical mechanical deformation activates endothelial and stromal mechanotransduction pathways, including eNOS and integrin-mediated signaling, that promote inflammation resolution, angiogenesis, fibroblast proliferation, extracellular matrix deposition, and collagen cross-linking [6] (Figure 1).

Humidification improves oxygen diffusion, maintains a moist wound environment, and supports organized tissue formation and durable healing [21]. Together, these mechanisms promote a biologically favorable wound environment that supports not only closure but also durable tissue repair [6].

## 3. Results

### 3.1. Evidence from Randomized Controlled Trials

High-quality randomized evidence supporting TWO_2_ therapy is provided by the multicenter, double-blind, sham-controlled TWO_2_ Study conducted in patients with chronic DFUs that had failed to respond to standard care and published in published in *Diabetes Care* [22]. Participants were randomized to receive either active TWO_2_ therapy or sham treatment in addition to optimal wound care.

At the first prespecified interim analysis, healing at 12 weeks was significantly higher in the active TWO_2_ group compared with sham (41.7% versus 13.5%), with an adjusted odds ratio of 6.00 (97.8% CI 1.44, 24.93), *p* = 0.004, after controlling for ulcer severity. Time-to-healing analysis demonstrated a more than fourfold greater likelihood of healing over 12 weeks in the TWO_2_ group (HR = 4.66 [97.8% CI 1.36, 15.98], *p* = 0.004).

Importantly, durability of healing was confirmed at one year, with a significant 56% of ulcers in the active group remaining closed compared with just 27% in the sham group (*p* = 0.013). This sustained healing at one year suggests improved tissue remodeling, which was further demonstrated by a sixfold reduced ulcer recurrence.

### 3.2. Evidence in Venous Leg Ulcers

In a prospective controlled study of 132 patients with chronic VLUs present for more than two years, TWO_2_ therapy was compared to conventional compression dressings [23,24]. Key and statistically significant findings:

- Healing rate: 76% vs. 46% at 12 weeks (*p* < 0.0001)
- Median time to closure: 57 vs. 107 days (*p* < 0.0001)
- Recurrence at 36 months: 6% vs. 47% (*p* < 0.0001)

Pain reduction and improved infection resolution were also observed in the TWO_2_ group. These findings extend the relevance of TWO_2_ beyond diabetic wounds to those with venous pathology, where edema and hypoxia are central features. This is particularly important as most patients treated in the real world are comorbid with multiple etiological traits.

### 3.3. Real-World Evidence in Chronic Lower Extremity Wounds

While randomized trials establish efficacy, real-world evidence is critical to understanding clinical value across heterogeneous, high-risk populations. A recent large cohort retrospective study by Lohr et al., evaluated the effectiveness of TWO_2_ in chronic lower extremity wounds of varying etiologies [25]. In this group of 3126 patients, 64.8% (*n* = 2027) achieved complete healing in 4.2 (SD ± 2.5) months, despite a mean pre-treatment wound age of 7 (±15.9) months. The need for retreatment due to wound recurrence was only 2.7% (*n* = 54), with a mean follow up time of 13.9 (±4.9) months and the rates of hospitalization and amputation were 3.7% (*n* = 115) and 6.1% (*n* = 191), respectively, substantially lower than historical standards [1,26,27,28].

Subgroup analyses demonstrated healing rates of 63.3% in a mean time of 4.2 (±2.5) months in DFUs, 72.1% in a mean time of 4 (±2.6) months in VLUs, 59.3% in a mean time of 3.9 (±2.5) months in arterial ulcers, and 65.6% in a mean time of 4 (±2.3) months in atypical wounds. Compared with large wound registry data, the more medically complex patients in the TWO_2_ study had higher rates of healing in both DFU and VLU (63% at 18 weeks vs. 45% at 20 weeks for DFU, and 72% at 17 weeks vs. 57% at 16 weeks for VLU) [29]. The findings of this large study further support the efficacy of TWO_2_ in chronic wounds of varied etiologies.

### 3.4. Real-World Comparative Effectiveness Evidence

Comparative effectiveness evidence is crucial to evidence-based clinical decision-making, empowering clinicians and patients to select treatments most likely to produce meaningful outcomes, and enabling health systems to direct resources toward interventions that demonstrate durable value. A retrospective cohort analysis of 202 patients by Yellin et al., evaluated the effectiveness of home-based TWO_2_ therapy compared to other advanced therapies in patients with DFUs treated at two U.S. Veterans Affairs hospitals [30].

#### 3.4.1. TWO_2_ vs. No TWO_2_ (Additive Model)

In this comparison, outcomes in patients who had received TWO_2_ therapy at any point during their care pathway (TWO_2_) versus those who had not received TWO_2_ therapy (NO TWO_2_) were evaluated. Patients in both groups may have received other additional advanced treatment interventions, therefore TWO_2_ therapy was considered an additive adjunctive treatment.

In unmatched cohorts, patients treated with TWO_2_ experienced 88% fewer hospitalizations (6.6% vs. 54.1%, *p* < 0.0001) and 71% fewer amputations (12.1% vs. 41.4%, *p* < 0.0001) over one year when compared with patients who did not receive TWO_2_. After propensity score matching for age, wound severity, comorbidities, prior amputation, and use of other advanced therapies, TWO_2_ therapy was associated with an 82% reduction in hospitalizations (7.1% vs. 40.0%, *p* < 0.0001) and a 73% reduction in amputations (8.6% vs. 31.4%, *p* = 0.0007), compared with no TWO_2_. Logistic regression demonstrated an almost ninefold greater risk of hospitalization and fivefold greater risk of amputation among patients who did not receive TWO_2_. These reductions persisted across Wagner grades and in patients with peripheral arterial disease, prior amputation, with end-stage renal disease (ESRD) and even on dialysis.

#### 3.4.2. TWO_2_ Only vs. Other Advanced Modalities (Direct Either/or Comparison)

A unique and clinically relevant aspect of the Yellin et al., analysis was a second comparison that directly evaluated TWO_2_ only against other advanced therapies (NPWT, skin substitutes, growth factors).

**Figure 2:**
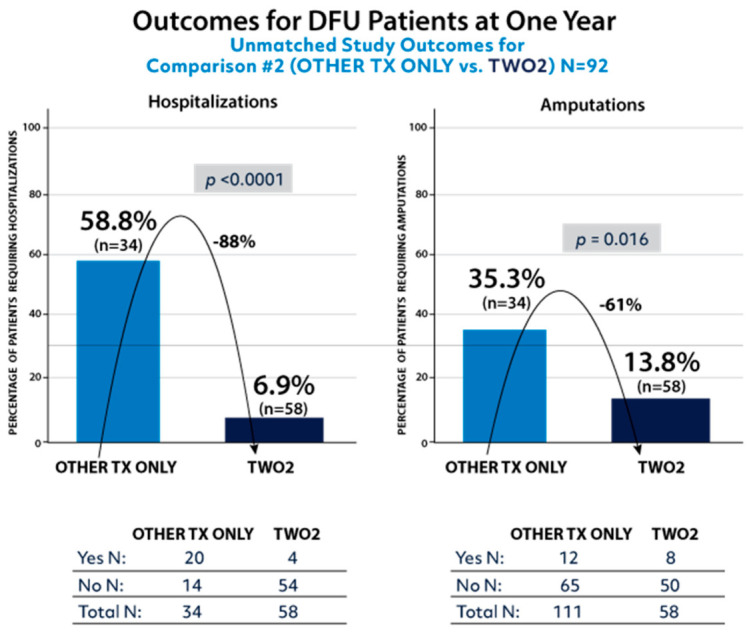
TWO_2_ Only vs. Other Advanced Modalities: One Year Outcomes: Patients treated with TWO_2_ alone experienced significantly better outcomes than those treated with only other advanced modalities, demonstrating an 88% relative reduction in hospitalization and 61% fewer amputations (Reprinted/adapted from ref. [30]).

Patients treated with TWO_2_ alone experienced significantly better outcomes than those treated with only other advanced modalities, demonstrating an 88% relative reduction in hospitalization (6.9% vs. 58.8%, *p* < 0.0001) and 61% fewer amputations (13.8% vs. 35.3%, *p* = 0.016) at one year (Figure 2).

These outcomes suggest that TWO_2_ therapy not only offers additive benefit but may also be associated with significantly better outcomes compared with other traditionally used advanced wound care modalities when used as a primary adjunctive therapy. Prospective head-to-head randomized controlled trials remain needed to establish causality.

In sum, the study demonstrated that the use of TWO_2_ therapy, with or without other adjunctive treatments, was associated with significantly reduced frequency of wound-related hospitalization and amputation for patients afflicted with DFUs. Given that both outcomes are associated with higher costs, increased healthcare utilization, and poorer quality of life, these findings highlight TWO_2_ therapy’s potential value as a cost-effective, patient-centered modality in DFU management [2,3,4].

## 4. Discussion

### 4.1. Contextualizing TWO_2_ Amongst Other Advanced Wound Technologies

The advanced wound care modalities in Table 1 each have defined indications and limitations relevant to chronic lower-extremity wound pathophysiology.

Hyperbaric oxygen therapy (HBOT) delivers 100% oxygen at 2.0 to 3.0 atmospheres absolute via systemic delivery, increasing dissolved plasma oxygen and tissue oxygen gradients. Cochrane evidence supports improved short-term healing in selected diabetic foot ulcers and refractory wounds [31]. Because delivery is systemic, efficacy depends on intact arterial inflow and microvascular perfusion, limiting use in severe peripheral arterial disease. Additional constraints include risks of barotrauma and oxygen toxicity, chamber availability, and patient suitability for hyperbaric exposure [31].

Continuous delivery topical oxygen (CDO) systems apply sustained low-flow, low-pressure oxygen (3 to 15 mL/h) under a sealed dressing. Controlled studies and a recent systematic review report improved healing in Wagner Grade 1 and 2 DFUs [32,33]. CDO does not reduce edema or restore lymphatic function, and the comparative evidence base remains smaller than for TWO_2_ therapy [32,33].

Negative pressure wound therapy (NPWT) applies subatmospheric pressure via a sealed dressing, promoting granulation, exudate removal, and angiogenic signaling. It is best supported for wound bed preparation, exudate management, and acute traumatic or surgical wounds [34]. NPWT does not directly correct hypoxia or restore limb hemodynamics; practical considerations include clinical staff requirements for application (typically 2–3x/week), periwound maceration, treatment-related pain, and bleeding risk.

Bioengineered skin substitutes provide an exogenous extracellular matrix scaffold, with some formulations incorporating viable cellular components or growth-factor enriched constructs. FDA-approved living skin equivalents have demonstrated improved healing of DFUs and VLUs that have failed initial standard care [35]. Effectiveness requires a clean, well-perfused, well-oxygenated wound bed and is reduced in hypoxic or protease-rich environments. Multiple applications are often needed at substantial cost, with outcomes dependent on patient selection and wound bed preparation [35].

Growth factor therapies, such as recombinant human platelet-derived growth factor (becaplermin), deliver exogenous peptide signaling for fibroblast recruitment, angiogenesis, and granulation. Becaplermin is FDA-approved as an adjunct for neuropathic DFUs. Growth factor activity is reduced by proteolytic degradation in chronic wound exudate and by tissue hypoxia [36].

Compression therapy applies sustained, graduated external pressure, reducing venous hypertension and supporting lymphatic drainage. It is the standard of care for venous leg ulcers, with consistent evidence for improved healing and reduced recurrence [37,38]. Compression does not directly correct tissue hypoxia or provide cellular reparative signaling. Adherence is a recognized challenge, and use is limited or contraindicated in significant arterial insufficiency [37].

Across these modalities, therapies targeting a single component of chronic wound pathology may improve intermediate outcomes, whereas therapies addressing multiple interdependent drivers of chronicity may offer a more favorable mechanistic profile for durable healing, consistent with the comparative outcomes detailed in Section 3. A structured comparison is presented in Table 1.

### 4.2. Practical Considerations and Clinical Integration

TWO_2_ therapy is designed for home-based administration by the patient, reducing treatment burden and improving adherence, while advancing access to care. It can be integrated into care pathways either as an adjunct to other advanced modalities or as an alternative when other therapies fail. Sequential strategies may include initial NPWT for exudate control followed by transition to TWO_2_ for angiogenesis and epithelialization, or concurrent use with skin substitutes to optimize graft uptake.

### 4.3. Cost-Effectiveness Considerations

The following economic considerations are derived from retrospective cohort analyses and a published decision-analytic Markov model; they represent modeled estimates and should be interpreted in that context rather than as findings from prospective comparative cost analyses. The original authors did not conduct an independent health economic evaluation as part of this review.

Wound recurrence, hospitalizations, and amputations represent major cost drivers in chronic wound care. Repeat emergency department admissions, surgical intervention, rehabilitation, prosthetics, and long-term disability contribute to the economic burden. Even more difficult to quantify are the negative psychosocial and quality-of-life impacts on patients and caregivers.

The reductions in hospitalization (up to 88%) and amputation (up to 73%) observed in real-world TWO_2_ cohorts suggest significant potential downstream cost savings. Additionally:

- Home-based therapy reduces outpatient visit frequency.
- Improved durability should reduce recurrence-related costs.
- Lower amputation rates should reduce lifetime disability expenditures.

A recent Markov model analysis suggested that TWO_2_ may be cost-dominant compared with standard care, with modeled estimates indicating lower total two-year costs and improved quality-adjusted life years (QALYs). These projections are based on modeling assumptions and require confirmation through prospective economic evaluations comparing TWO_2_ directly with NPWT, HBOT, and skin substitutes across longer time horizons [2].

### 4.4. Limitations

The evidence base for TWO_2_ therapy includes both randomized and observational data, each with inherent limitations. Even though largely compensated for by robust study designs and statistical approaches such as propensity scoring, retrospective analyses may be subject to unmeasured confounding, while randomized trials may not fully capture real-world complexity. Direct head-to-head randomized comparisons with NPWT, HBOT, or bioengineered skin substitutes are currently lacking and represent a priority gap for future research. Additionally, the available comparative literature does not provide stratified efficacy analyses by wound chronicity, severity grade, or comorbidity burden, and the variable follow-up duration in the Lohr cohort (13.9 ± 4.9 months) limits direct interpretation of recurrence and hospitalization endpoints. The cost-effectiveness data summarized here derive from a published decision-analytic Markov model and do not constitute prospective economic evidence. Despite these limitations, the consistency of findings and long-term outcomes across study designs and wound types is supportive of the observed therapeutic benefits.

## 5. Conclusions

TWO_2_ therapy is supported by a converging body of evidence demonstrating improved healing durability and substantial reductions in hospitalization and amputation in patients with multimorbid chronic lower-extremity wounds. The randomized TWO_2_ Study establishes efficacy, while the real-world analysis by Yellin and colleagues demonstrates generalizability in far broader comorbid patient populations, as well as comparative effectiveness against other advanced wound modalities. The large cohort retrospective study by Lohr and colleagues further supports the generalizability of healing outcomes across varied lower extremity wound types, and in populations refractory to standard care and advanced therapies. By addressing hypoxia, edema, and impaired microcirculation simultaneously, TWO_2_ therapy offers a mechanism-driven approach that translates into meaningful patient-centered outcomes [6,22,23,30].

These findings support the earlier and broader adoption of TWO_2_ therapy in the management of chronic lower extremity wounds, with the potential to reduce wound recurrence, lower amputation risk, and decrease the economic burden and social toll of chronic wounds on patients, caregivers, and the healthcare system.

## Figures and Tables

**Table 1:** Relative Comparison of Advanced Wound Therapies.

Therapy	pO_2_ Increase	Edema Reduction/ Lymphatic Activation	Inflammation Resolution	Angiogenesis Upregulation	Bioburden Control	ECM/ Cellular Repair	Unique Mechanism(s)
TWO_2_ [6,22]	+++	+++	+++	+++	++/+++	+++	Pressurized topical oxygen with non-contact cyclical compression; concurrent modulation of hypoxia, edema, and mechanotransduction signaling pathways
HBOT ** [31]	+++	+ *	++	++	++	++	Systemic hyperoxygenation increasing dissolved plasma oxygen and tissue oxygen gradients
Continuous Delivery Oxygen [32,33]	++	−	++	++	++	++	Sustained low-flow topical oxygen diffusion maintaining continuous wound surface oxygen gradient
NPWT [34]	+ *	+	++	++	+	++	Macro/micro deformation induced mechanotransduction with controlled exudate removal and wound edge stabilization
Skin Substitutes [35]	−	−	+	++	−	+++	Bioactive extracellular matrix scaffold providing structural support, cellular signaling, and growth factor modulation
Growth Factors [36]	−	−	+	++	−	++	Exogenous peptide-mediated activation of cell proliferation and angiogenic signaling pathways
Compression [37,38]	+ *	+++	++	+	+	+	Venous hypertension reduction and lymphatic unloading through sustained, graduated external pressure

Relative Comparison of Advanced Wound Therapies: Ratings reflect the authors’ qualitative synthesis of dominant direct mechanisms reported in published mechanistic and clinical literature. “+++” denotes primary, robust engagement of the pathway supported by multiple published mechanistic and clinical studies; “++” denotes a consistent secondary contribution supported by published evidence; “+” denotes an indirect or context-dependent contribution; “−” denotes no established primary direct mechanism. Ratings are comparative synthesis tools and not absolute measures of clinical efficacy or relative effectiveness. * Indirect effects mediated via perfusion or edema modulation rather than direct oxygen delivery or antimicrobial action. ** Requires intact vasculature.

## Data Availability

No new data were created or analyzed in this study.

## References

[1] Sen C.K. (2023). Human Wound and Its Burden: Updated 2022 Compendium of Estimates. Adv. Wound Care.

[2] Kerr M., Wild D., Edmonds M., Boulton A.J.M. (2025). Cost effectiveness of topical wound oxygen therapy for chronic diabetic foot ulcers. J. Diabetes Complicat..

[3] Rice J.B., Desai U., Cummings A.K.G., Birnbaum H.G., Skornicki M., Parsons N.B. (2014). Burden of diabetic foot ulcers for medicare and private insurers. Diabetes Care.

[4] Margolis D.J., Malay D.S., Hoffstad O.J., Leonard C.E., MaCurdy T., Tan Y., Molina T., de Nava K.L., Siegel K.L. (2011). Economic burden of diabetic foot ulcers and amputations. Data Points Publication Series [Internet].

[5] Sen C.K., Gordillo G.M., Roy S., Kirsner R., Lambert L., Hunt T.K., Gottrup F., Gurtner G.C., Longaker M.T. (2009). Human skin wounds: A major and snowballing threat to public health and the economy. Wound Repair Regen..

[6] Lohr J.M., Raffetto J.D., Dexter D.J., Regulski M.J., Edmonds M.E., Ozsvath K.J., Blakely M.M. (2026). A synergistic multimodality treatment approach to address the key drivers of wound chronicity. J. Vasc. Surg. Venous Lymphat. Disord..

[7] Zhao R., Liang H., Clarke E., Jackson C., Xue M. (2016). Inflammation in Chronic Wounds. Int. J. Mol. Sci..

[8] Mustoe T.A., O’Shaughnessy K., Kloeters O. (2006). Chronic Wound Pathogenesis and Current Treatment Strategies: A Unifying Hypothesis. Plast. Reconstr. Surg..

[9] Frykberg R.G., Banks J. (2015). Challenges in the Treatment of Chronic Wounds. Adv. Wound Care.

[10] Qi X., Xiang Y., Li Y., Wang J., Chen Y., Lan Y., Liu J., Shen J. (2025). An ATP-activated spatiotemporally controlled hydrogel prodrug system for treating multidrug-resistant bacteria-infected pressure ulcers. Bioact. Mater..

[11] Zhang H., Liang Q., Ji Y., Chen Q., Jiang W., Zhang D., Wu Y., Yu L., Chen W., Liu R. (2025). Facile fabrication of antioxidative and antibacterial hydrogel films to accelerate infected diabetic wound healing. Bioact. Mater..

[12] Sen C.K. (2009). Wound healing essentials: Let there be oxygen. Wound Repair Regen..

[13] Tuckey B., Srbely J., Rigney G., Vythilingam M., Shah J. (2021). Impaired Lymphatic Drainage and Interstitial Inflammatory Stasis in Chronic Musculoskeletal and Idiopathic Pain Syndromes: Exploring a Novel Mechanism. Front. Pain Res..

[14] Schwager S., Detmar M. (2019). Inflammation and Lymphatic Function. Front. Immunol..

[15] Fries R.B., Wallace W.A., Roy S., Kuppusamy P., Bergdall V., Gordillo G.M., Melvin W.S., Sen C.K. (2005). Dermal excisional wound healing in pigs following treatment with topically applied pure oxygen. Mutat. Res. Fundam. Mol. Mech. Mutagen..

[16] Gottrup F., Dissemond J., Baines C., Frykberg R., Jensen P.Ø., Kot J., Kröger K., Longobardi P. (2017). Use of Oxygen Therapies in Wound Healing. J. Wound Care.

[17] Frykberg R., Andersen C., Chadwick P., Haser P., Janssen S., Lee A., Niezgoda J., Serena T., Stang D., Agarwal A. (2023). Use of Topical Oxygen Therapy in Wound Healing. J. Wound Care.

[18] Chettouh-Hammas N., Grillon C. (2024). Physiological skin oxygen levels: An important criterion for skin cell functionality and therapeutic approaches. Free Radic. Biol. Med..

[19] Oropallo A.R., Serena T.E., Armstrong D.G., Niederauer M.Q. (2021). Molecular biomarkers of oxygen therapy in patients with diabetic foot ulcers. Biomolecules.

[20] Arsenault K.A., McDonald J., Devereaux P.J., Thorlund K., Tittley J.G., Whitlock R.P. (2011). The use of transcutaneous oximetry to predict complications of chronic wound healing: A systematic review and meta-analysis. Wound Repair Regen..

[21] Nuutila K., Eriksson E. (2021). Moist Wound Healing with Commonly Available Dressings. Adv. Wound Care.

[22] Frykberg R.G., Franks P.J., Edmonds M., Brantley J.N., Téot L., Wild T., Garoufalis M.G., Lee A.M., Thompson J.A., Reach G. (2020). A Multinational, Multicenter, Randomized, Double-Blinded, Placebo-Controlled Trial to Evaluate the Efficacy of Cyclical Topical Wound Oxygen (TWO_2_) Therapy in the Treatment of Chronic Diabetic Foot Ulcers: The TWO_2_ Study. Diabetes Care.

[23] Tawfick W.A., Sultan S. (2013). Technical and Clinical Outcome of Topical Wound Oxygen in Comparison to Conventional Compression Dressings in the Management of Refractory Nonhealing Venous Ulcers. Vasc. Endovasc. Surg..

[24] Tawfick W., Sultan S. (2009). Does Topical Wound Oxygen (TWO_2_) Offer an Improved Outcome over Conventional Compression Dressings (CCD) in the Management of Refractory Venous Ulcers (RVU)? A Parallel Observational Comparative Study. Eur. J. Vasc. Endovasc. Surg..

[25] Lohr J.M., Boulton A.J.M., Hingorani A., Shaw P.M., Rowe V.L., Rogers L.C., Dua A. (2026). Retrospective Review of 3126 Patients with Chronic Lower Extremity Wounds Treated with Intermittent Topical Oxygen Therapy. JVS-Vasc. Insights.

[26] Armstrong D.G., Boulton A.J.M., Bus S.A. (2017). Diabetic Foot Ulcers and Their Recurrence. N. Engl. J. Med..

[27] McDaniel H.B., Marston W.A., Farber M.A., Mendes R.R., Owens L.V., Young M.L., Daniel P.F., Keagy B.A. (2002). Recurrence of chronic venous ulcers on the basis of clinical, etiologic, anatomic, and pathophysiologic criteria and air plethysmography. J. Vasc. Surg..

[28] McDermott K., Fang M., Boulton A.J.M., Selvin E., Hicks C.W. (2023). Etiology, Epidemiology, and Disparities in the Burden of Diabetic Foot Ulcers. Diabetes Care.

[29] Fife C.E., Eckert K.A., Carter M.J. (2018). Publicly Reported Wound Healing Rates: The Fantasy and the Reality. Adv. Wound Care.

[30] Yellin J.I., Gaebler J.A., Zhou F.F., Niecko T., Novins O., Ockert A., Krzynowek D., Garoufalis M.G., Lee A.M., Frykberg R.G. (2022). Reduced Hospitalizations and Amputations in Patients with Diabetic Foot Ulcers Treated with Cyclical Pressurized Topical Wound Oxygen Therapy: Real-World Outcomes. Adv. Wound Care.

[31] Kranke P., Bennett M.H., Martyn-St James M., Schnabel A., Debus S.E., Weibel S. (2015). Hyperbaric oxygen therapy for chronic wounds. Cochrane Database Syst. Rev..

[32] Gordillo G.M., Roy S., Khanna S., Schlanger R., Khandelwal S., Phillips G., Sen C.K. (2008). Topical oxygen therapy induces vascular endothelial growth factor expression and improves closure of clinically presented chronic wounds. Clin. Exp. Pharmacol. Physiol..

[33] Nagarsheth K., Kankaria A., Marsella J., Dunlap E., Hawkins S., Ucuzian A., Lal B.K. (2024). Systematic review of the effects of topical oxygen therapy on wound healing. JVS-Vasc. Insights.

[34] Huang C., Leavitt T., Bayer L.R., Orgill D.P. (2014). Effect of negative pressure wound therapy on wound healing. Curr. Probl. Surg..

[35] Vecin N.M., Kirsner R.S. (2023). Skin substitutes as treatment for chronic wounds: Current and future directions. Front. Med..

[36] Yamakawa S., Hayashida K. (2019). Advances in surgical applications of growth factors for wound healing. Burn. Trauma.

[37] Partsch H., Mortimer P. (2015). Compression for leg wounds. Br. J. Dermatol..

[38] Partsch H. (2012). Compression Therapy: Clinical and Experimental Evidence. Ann. Vasc. Dis..

